# Age independency of mobility decrease assessed using the Locomotive Syndrome Risk Test in elderly with disability: a cross-sectional study

**DOI:** 10.1186/s12877-017-0698-7

**Published:** 2018-01-26

**Authors:** Keiko Yamada, Shingo Muranaga, Tomohiro Shinozaki, Kozo Nakamura, Sakae Tanaka, Toru Ogata

**Affiliations:** 1 Departments of Sensory & Motor System Medicine, Faculty of Medicine, The University of Tokyo, 7-3-1, Hongo, Bunkyo-ku, Tokyo, Japan; 2 Kameda Medical Center, 929 Higashi-cho, Kamogawa City, Chiba Prefecture Japan; 3 Department of Biostatistics, School of Public Health, The University of Tokyo, 7-3-1, Hongo, Bunkyo-ku, Tokyo, Japan; 4 Department of Rehabilitation for Movement Functions, Research Institute, National Rehabilitation Center for Persons with Disabilities, 4-1, Namiki, Saitama, 359-8555 Japan

**Keywords:** Mobility decrease, Age-independent, Care level, Disabled elderly, Cross-sectional study, Locomotive syndrome

## Abstract

**Background:**

Mobility decrease is reportedly age-dependent in community dwelling elderly, and a major factor of disability in the geriatric population. The purpose of this study is to examine whether mobility decrease, as assessed using a set of tests, is similarly age-dependent in elderly adults who already have disability.

**Methods:**

One hundred thirty-five community-dwelling elderly (54 men, 81 women) with disability and 1469 independent community dwellers (1009 men, 460 women) were analyzed. Disability was defined having a certified need for care under the long-term care insurance system in Japan. Lower extremity mobility decrease was quantified using the Locomotive Syndrome Risk Test, which comprises the two-step test, stand-up test, and 25-Question Geriatric Locomotive Function Scale (GLFS-25).

**Results:**

Multivariable regression analyses indicated no age-related decrease in the three test scores among elderly with disability, whereas these scores all decreased with age among independent community dwellers. All the test scores decreased as care level increased.

**Conclusions:**

Mobility decrease among elderly adults with disability is unrelated to age. However, the severity of care level is associated with mobility decrease.

## Background

Japan is now a super-aged society, and the rapidly increasing number of disabled elderly and expanding financial burden that accompanies this status has become an urgent issue [1]. As a measure against this situation, the Japanese Government launched the national long-term care insurance (LTCI) system in 2000, with the goal of providing suitable individual care services for elderly with disability [2]. Additionally, the Japanese Orthopaedic Association (JOA) proposed the concept of “locomotive syndrome” in 2007, which they defined as a condition of decreased mobility in activities essential to daily life, such as walking, standing up, and climbing stairs, resulting from impairment in the locomotive organs [3–5]. They proposed this concept because one of the top reasons for LTCI certification is musculoskeletal diseases [3, 4, 6–9]. This concept also aimed to increase public awareness of mobility decrease and its management strategies by clarifying the risk factors, which is especially necessary because mobility is known to begin decreasing early on in life [3, 8, 10]. In 2013, to quantify lower extremity mobility among all ages, the JOA proposed the Locomotive Syndrome Risk Test that comprises three tests: the two-step test, stand-up test, and the 25-Question Geriatric Locomotive Function Scale (GLFS-25) [9, 11–13]. This test is expected to be a useful screening tool for detecting mobility decrease in middle-aged and elderly adults because of its simplicity and feasibility.

As noted above, mobility decrease is age-dependent; it can even begin early in adulthood and represents a key driving force of disability, which in turn is associated with a high mortality risk in the geriatric population [6, 10, 14–16]. However, it remains unclear whether mobility decrease among elderly adults who already have disability is also age-dependent. It would be necessary to elucidate the patterns of mobility decrease in this vulnerable population in order to ensure proper intervention.

Thus, the purpose of the present study is to examine whether mobility decrease in already disabled elderly adults is age-dependent, using the Locomotive Syndrome Risk Test to quantify mobility decrease. We also investigated the relationship between severity of care level in elderly with disability and mobility decrease because, although previous research has indicated that disability, especially that related to lower extremity mobility, is a strong predictor of mortality [14, 16], the relationship between quantified mobility decrease and severity of disability has yet not been established.

## Methods

### Subjects

From 2012 to 2016, 135 community-dwelling elderly (54 men, 81 women) from Kamogawa city, Chiba prefecture, Japan and who were certified as needing care under the LTCI system were examined as participants with disability. Furthermore, 1469 independent community dwellers (1009 men, 460 women) aged 40–89 years, were also recruited when they visited a medical center for a periodic medical check-up in the same city. These visitors were evaluated by physiotherapists or physicians through general medical examination, such as visual inspection and medical interview; in this study, we included only those subjects without apparent mobility limitation. All participants completed the Locomotive Syndrome Risk Test. All the measurements were performed as a part of the regular services in each setting, and the data were reviewed retrospectively. The utilization of these data for the research purpose was approved by the ethics committees of Kameda Medical Center.

### Certification of care needs in the Japanese long-term care insurance system

We defined disability as being certified as “requiring care” under the LTCI system in Japan. The Japanese LTCI system divides elderly adults into groups of lighter and heavier care—specifically, as requiring support (support-level) or long-term care (care-level) in their activities of daily living, respectively [2]. The standards for LTCI certification are uniformly and objectively applied throughout Japan [17]. The level of LTCI certification has been reported to be well correlated with the Barthel Index [18]. The detailed process of assessing eligibility for the certification of needed care has been described in detail elsewhere [6, 19, 20]. Briefly, an elderly adult requiring help with ADLs applies for certification of care needs to their municipal government. A professional board in each municipal government determines the certification and its level based on the elderly adult’s primary physician’s opinion and a nationally standardized score calculated from the results of a 82-item, interviewer-administered questionnaire assessing ADLs, mental status, and medical activities [6, 19, 20].

### Locomotive Syndrome Risk Test

To assess mobility decrease, we used a set of three tests assessing Locomotive Syndrome proposed by the JOA: the two-step test, stand-up test, and GLFS-25 [11–13]. The two-step test and the stand-up test are functional tests while the GLFS-25 is a self-administered questionnaire that evaluates motor dysfunction. The validity, reliability, and feasibility of all three tests have been confirmed [13, 21, 22]. We provide summaries of the three tests below; for those interested, detailed descriptions of these tests are available in other studies [11–13, 21, 22].

### Two-step test

This test measures the maximum stride length of two steps from the standing posture. Participants are told to exercise caution so as not to lose balance. The two-step test score is a standardized value calculated by the following formula: length of the two steps (cm) ÷ the subject’s height (cm). The two-step test score reportedly has a strong correlation with maximum walking speed [11].

### Stand-up test

This test assesses participants’ leg strength by having them stand up, using one or both legs, from a specified height and maintain their posture. Subjects are requested to stand from four different height stools (10, 20, 30, and 40 cm) with one or both legs. If a subject succeeds in standing up and maintaining that posture for 3 s, the trial is judged as completed. Participants are allocated a score of 0–8 based on their performance, as shown in Table 1. Higher scores indicate a better ability to stand up. A previous study has shown that there is a significant correlation between the stand-up test score and weight-bearing index: an indicator of lower extremity strength, which is calculated by normalizing the knee extensor strength divided by body weight [12, 23].

**Table 1:** Scoring system of stand-up test

	Two-leg stand					One-leg stand			
Height	Fail at 40 cm	40 cm	30 cm	20 cm	10 cm	40 cm	30 cm	20 cm	10 cm
Score	0	1	2	3	4	5	6	7	8

One-leg stand requires subjects to succeed at indicated height in both *right* and *left* leg

### 25-question geriatric locomotive function scale

This self-administered questionnaire is used to evaluate motor dysfunction. It comprises 25 items relating to pain, activities of daily living, social functions, and mental health status; each is scored on a range of 0–4. The total score ranges from 0 to 100, with higher scores indicating worse locomotive condition.

### Statistical analysis

Pearson’s correlation coefficient or Spearman’s rank correlation coefficient were calculated to measure the associations of the three scales with age and sex. Then, to quantify the dependencies of the three test scores on age and sex, we performed multivariable regression analyses. The support- and care-level subgroups were combined in the regression analysis as the “certified level groups” because the correlations between the three test scores and age had the same tendency in both subgroups. We further examined the differences in the coefficients of independent community dwellers and the elderly with disability via an interaction test, to determine whether the effects of age/sex on the test scores differed between these two groups. In the model we included the main effects of age, sex, and group (independent community dwellers vs. the elderly with disability), as well as the age- and sex- product terms with group. We further adjusted for support and care level in the certified elderly group. *P*-values for the product terms measure the differences between the coefficients in the certified elderly and independent community dwellers (i.e., *p*-values for interaction tests). Differences in the scores between the different age categories in independent community dwellers were assessed using analysis of variance with Tukey’s tests (for the two-step test) or the Kruskal-Wallis H test with Bonferroni corrections (for the stand-up test and GLFS-25). Elderly individuals with disability were not assessed in terms of age categories because we did not observe age dependency in that group. A p-value of <0.05 was taken to indicate statistical significance. All statistical analyses were performed using IBM SPSS 22.0 (IBM Corp., Armonk, NY, US).

## Results

### Background characteristics and the relationship of severity of care level and three test scores

**Fig. 1:**
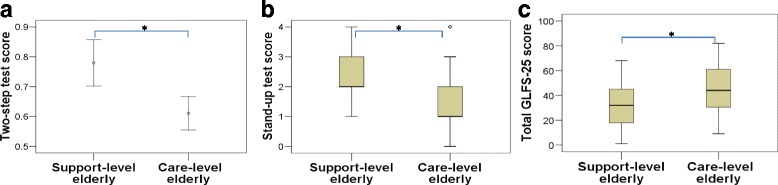
Three scores on the Locomotive Syndrome Risk Test for elderly with disability. The scores for the (**a**) two-step test, (**b**) stand-up test, and (**c**) total GLFS-25 were significantly different between the support- and care-level elderly with disability (*p* < 0.001). The mean and 95% CI of the two-step score is shown in (**a**); the median, interquartile range, and extreme cases of the stand-up test score and total GLFS-25 score are shown in (**b**) and (**c**). *p* < 0.05; *t*-test was used for two-step test score and the Mann-Whitney U test for stand-up test/total GLFS-25 score in order to compare the support- and care-level groups

Table 2 shows the background characteristics of the subjects. Among the 135 individuals with certified disability, the mean age (± standard deviation) was 82.6 ± 5.0 years, while among the 1469 independent community dwellers, the mean age was 62.2 ± 8.5 years. We observed no difference in the mean age between the elderly in the support- and care-level subgroups. However, the three scores of the tests were significantly worse in care-level subjects than in the support-level subjects (*p* < 0.001) (Fig. 1).

**Table 2:** Background characteristics of subjects and descriptive statistics of three test scores

	Certified elderly under the LTCI system (elderly with disability)				Independent community dwellers
	Total *n* = 135	Support-level elderly *n* = 40	Care-level elderly *n* = 95	*P* value	Total *n* = 1469
Age (yrs)	82.6 ± 5.0	82.0 ± 5.7	82.7 ± 7.2	0.592	62.2 ± 8.5
Sex (males)	54 (40.0)	14 (35.0)	40 (42.1)	0.445	1009 (68.6)
Two-step test score	0.66 (0.27)	0.78 (0.24)*	0.61 (0.27)*	0.001	1.53 (0.13)
Stand-up test score	1.93 (1.13)	2.50 (0.93)*	1.69 (1.13)*	<0.001	4.81 (1.10)
Total GLFS-25 score^a^	41.6 (18.4)	33.8 (17.1)*	45.0 (18.0)*	0.001	6.64 (7.45)

Values are presented as n (%) or mean ± SD

*Statistically significant at the *p*<0.05 level; *t*-test was used for two-step test score and the Mann-Whitney U test for stand-up test/total GLFS-25 score in order to compare the support- and care-level groups; ^a^Total GLFS-25 score (*n* = 132) care-level elderly (*n* = 92); *SD* Standard deviation, *LTCI* Long-term care insurance, *GLFS* Geriatric Locomotive Function Scale

### Correlations between the three test scores and age in the disability subgroups

We observed no significant correlations between age and the three test scores in elderly with disability (Tables 3 and 4). On the other hand, the correlations between the three test scores and age were significant in independent community dwellers (*p* < 0.001; Table 5). The correlations among the three scales were significant in all three groups (support-level, care-level, and independent community dwellers), except for that between stand-up test score and total GLFS-25 score among support-level elderly (Tables 3, 4 and 5).

**Table 3:** Correlations between the three test scores and age in the support-level elderly (*n* = 40)

		Age	Two-step test score	Total GLFS-25 score	Stand-up test score	Sex
Age	Pearson’s r		−0.242	−0.054	−0.179	
	*P*-value		0.132	0.740	0.270	
Two-step test score	Pearson’s r			*−0.424* ^*^	*0.325* ^*^	
	*P*-value			0.006	0.041	
Total GLFS-25 score	Pearson’s r				−0.175	
	*P*-value				0.279	
Stand-up test score	Pearson’s r					
	*P*-value					
Sex	Spearman’s rho	−0.002	−0.037	−0.016	0.297	
	*P*-value	0.989	0.823	0.992	0.062	

*p < 0.05 two-tailed; Statistically significant correlations at the *p* < 0.05 level were italicized; *Pearson’s r* Pearson’s correlation coefficient, *Spearman’s rho* Spearman’s rank correlation coefficient, *GLFS* Geriatric Locomotive Function Scale

**Table 4:** Correlations between the three test scores and age in the care-level elderly (*n* = 95)^a^

		Age	Two-step test score	Total GLFS-25 score	Stand-up test score	Sex
Age	Pearson’s r		−0.066	−0.122	− 0.016	
	*P*-value		0.523	0.247	0.878	
Two-step test score	Pearson’s r			*−0.315* ^*^	*0.461* ^*^	
	*P*-value			0.002	< 0.001	
Total GLFS-25 score	Pearson’s r				*−0.302* ^*^	
	*P*-value				0.003	
Stand-up test score	Pearson’s r					
	*P*-value					
Sex	Spearman’s rho	*−0.323* ^*^	−0.103	− 0.027	*0.294* ^*^	
	*P*-value	0.001	0.318	0.799	0.004	

*p < 0.05, two-tailed; Statistically significant correlations at the *p* < 0.05 level were italicized; ^a^Total GLFS-25 score (*n* = 92); *Pearson’s r* Pearson’s correlation coefficient, *Spearman’s rho* Spearman’s rank correlation coefficient, *GLFS* Geriatric Locomotive Function Scale

**Table 5:** Correlations between the three test scores, age, and sex among independent community dwellers (*n* = 1469)

		Age	Two-step test score	Total GLFS-25 score	Stand-up test score	Sex
Age	Pearson’s r		*−0.356* ^*^	*0.190* ^*^	*−0.329* ^*^	
	*P*-value		< 0.001	< 0.001	< 0.001	
Two-step test score	Pearson’s r			*−0.288* ^*^	*0.427* ^*^	
	*P*-value			< 0.001	< 0.001	
Total GLFS-25 score	Pearson’s r				*−0.256* ^*^	
	*P*-value				< 0.001	
Stand-up test score	Pearson’s r					
	*P*-value					
Sex	Spearman’s rho	0.007	*0.240* ^*^	*−0.090* ^*^	*0.131* ^*^	
	*P*-value	0.799	< 0.001	0.001	< 0.001	

*p < 0.05, two-tailed; Statistically significant correlations at the *p* < 0.05 level were italicized; *Pearson’s r* Pearson’s correlation coefficient, *Spearman’s rho* Spearman’s rank correlation coefficient, *GLFS* Geriatric Locomotive Function Scale

### Multivariate regression analyses

Among elderly with disability, the multivariate regression analyses showed no age-related decrease; however, there was a decrease in all three test scores with increasing care level (*p* < 0.001; Table 6 left). On the other hand, among independent community dwellers, we found an age-related decrease in the three test scores (*p* < 0.001; Table 6 right). The interaction effects of disabled elderly/independent community dwellers and age were significant (Table 6).

**Table 6:** Multivariate regression analyses for the three test scores among elderly with disability and independent community dwellers

	Elderly with disability			Independent community dwellers				
Outcomes and covariates	β (Standardized β)	95%CI	*P*-value	β (Standardized β)		95%CI	*P*-value	*P* (interaction)
Two-step test score								
*Age*	−0.001 (−0.027)	−0.008; 0.006	0.752	−0.006 (−0.355)*		−0.006; −0.005	<0.001	0.015
*Sex*	−0.057 (−0.102)	−0.153; 0.038	0.238	0.067 (0.229)*		0.053; 0.080	<0.001	<0.001
*Support* vs. *care level*	−0.164 (0.272)*	−0.264; −0.064	0.001					
Stand-up test score								
*Age*	0.006 (0.035)	−0.021; 0.033	0.674	−0.043 (−0.328)*		−0.049; −0.036	<0.001	<0.001
*Sex*	0.583 (0.253)*	0.208; 0.958	0.003	0.274 (0.116)*		0.160; 0.388	<0.001	0.115
*Support* vs. *care level*	−0.851 (−0.344)*	−1,243; −0.459	<0.001					
Total GLFS-25 score^†^								
*Age*	−0.304 (−0.111)	−0.771; 0.163	0.200	0.167 (0.189)*		0.123; 0.211	<0.001	<0.001
*Sex*	−1.946 (−0.049)	−8.264; 4.573	0.570	−0.871 (−0.054)*		−1.678; −0.064	0.034	0.554
*Support* vs. *care level*	11.48 (0.287)*	4.772; 18.18	0.001					

^*^P < 0.05; Statistically significant regression coefficients at the *p*<0.05 level were italicized; †Total GLFS-25 score (*n* = 132); *β* Regression coefficient, *CI* Confidence interval, *GLFS* Geriatric Locomotive Function Scale

### Age-dependency of mobility in independent community dwellers

**Fig. 2:**
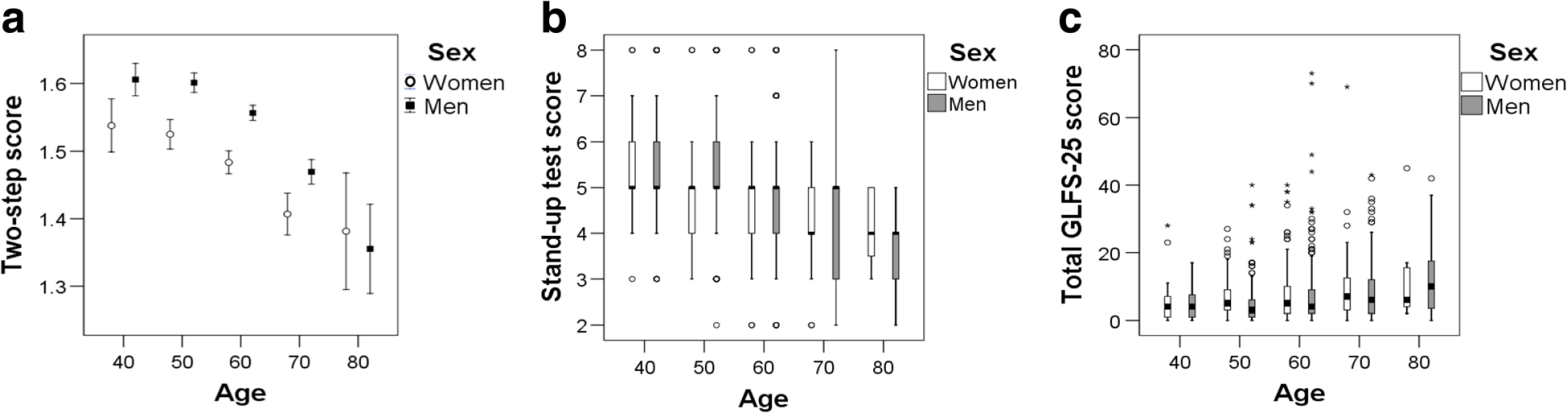
Three scores on the Locomotive Syndrome Risk Test in independent community dwellers. The scores for the (**a**) two-step test, (**b**) stand-up test, and (**c**) total GLFS-25 scores deteriorated with age. The mean and 95% CI are shown in (**a**); the median, interquartile range, and extreme cases of the stand-up test score and total GLFS-25 score are shown in (**b**) and (**c**)

Among independent community dwellers, all three test scores showed decreases with age. The three test scores among the independent community dwellers (grouped by age in 10-year increments) are shown in Table 7, and the trends are shown in Fig. 2a–c. The two-step test scores of subjects in their 60s and above were significantly lower among both men and women (*p* < 0.05; Table 7) than individuals younger than 60. Furthermore, the stand-up test scores of men in their 50s and above tended to be significantly worse than those below this age. Among women, the stand-up test scores were significantly lower only among those in their 70s (*p* < 0.005), but subjects in their 60s also showed comparatively lower scores (Table 7). Finally, the total GLFS-25 scores of men in their 60s and above tended to be significantly worse than those of younger individuals (*p* < 0.005). Among women, there were no significant differences by age, although subjects in their 70s and above tended to have worse total GLFS-25 scores (Table 7).

**Table 7:** Three test scores in independent community dwellers

Age strata (yrs)	n	Age, Mean(SD) (yrs)	Two-step test score, Mean(SD)	95%CI	Stand-up test score, Median	Total GLFS-25 score, Median
Men						
40–49	88	44.7(2.6)	1.60(0.11)	1.58–1.62	5	4
50–59	259	55.6(3.0)	1.60(0.11)	1.58–1.61	5^A^	3
60–69	458	63.9(2.6)	1.55(0.11)^ab^	1.54–1.56	5^AB^	4^B^
70–79	185	73.0(2.5)	1.46(0.12)^a^^bc^	1.45–1.48	5^ABC^	6^AB^
80–89	19	81.7(1.7)	1.35(0.13)^a^^bcd^	1.28–1.42	4^ABC^	10^AB^
Total	1009	62.1(8.7)	1.55(0.13)	1.54–1.56		
Woman						
40–49	33	45.7(2.5)	1.53(0.11)	1.49–1.57	5	4
50–59	118	55.8(2.7)	1.52(0.11)	1.50–1.54	5	4
60–69	237	63.9(2.7)	1.48(0.13)^b^	1.46–1.50	5^†^	5
70–79	64	73.4(2.8)	1.40(0.12)^abc^	1.37–1.43	4^ABC^	7^‡^
80–89	8	82.0(2.8)	1.38(0.10)^ab^	1.29–1.46	4	6
Total	460	62.2(7.9)	1.48(0.13)	1.47–1.49		

*SD* Standard deviation, *CI* Confidence interval, *GLFS* Geriatric locomotive function scale

^a^Significantly different (*p* < 0.05) from values of those aged in their 40s

^b^Significantly different (*p* < 0.05) from values of those aged in their 50s

^c^Significantly different (*p* < 0.05) from values of those aged in their 60s

^d^Significantly different (*p* < 0.05) from values of those aged in their 70s

^A^Significantly different (*p* < 0.005) from values of those aged in their 40s

^B^Significantly different (*p* < 0.005) from values of those aged in their 50s

^C^Significantly different (*p* < 0.005) from values of those aged in their 60s

^D^Significantly different (*p* < 0.005) from values of those aged in their 70s

^†^40s–60s 0.005 50s–60s 0.006 ^‡^40s–70s 0.007

## Discussion

In this study, we investigated mobility decrease among community-dwelling elderly with disability and independent community dwellers, using the highly feasible Locomotive Syndrome Risk Test. Among the elderly with disability, the scores of all three tests were unrelated to age; by contrast, among independent community dwellers, we found a negative relationship between the scores and age. We also found that all three test scores worsened as care level increased in elderly with disability. As noted above, mobility decrease has been demonstrated to be age-dependent and is a driving force for disability [6, 10, 14–16, 20–22, 24]. Specifically in Japan, increased age has been reported to be a predictor of LTCI certification [6, 20]. However, until this study, there was little understanding of the age-dependency of mobility decrease in elderly who already have disability. Moreover, although disability of lower extremity mobility reportedly predicts all-cause mortality risk well among the elderly [14, 16], the relationship between quantified mobility decrease and care level under the LTCI (which is demonstrably well-correlated with disability level) has not been established. Thus, we also investigated quantified mobility decrease according to care level among elderly with disability.

Our findings indicate that increased age is not related to a decrease in the three mobility test scores among elderly with disability. Mobility decrease has been shown to accelerate later in life, especially in age 70 or above, and has an extremely heterogeneous time course across individuals [25]. Our findings indeed suggest that there is a wide range of mobility levels and various trajectories in mobility decrease among elderly with disability. This result might be related to the severe complications often present in this vulnerable population [14, 19, 26]. Intervention strategies for elderly with disability should thus be based on evaluations of individual mobility, regardless of their age, given the various trajectories of mobility decrease. In contrast, we found the age-dependent decrease in mobility among the independent community dwellers that has been found in previous studies [21, 22]. This study was a relatively large-scale cross-sectional study of the healthy population, and thus provides reliable age reference data for future studies.

We further found that all three test scores decreased as care level increased. This accords with past findings indicating that mobility (as assessed with the Barthel Index) decreased as disability level under the LTCI increased [19, 26]. However, in these studies, the evaluation of lower extremity mobility was not clarified, despite its importance. Thus, we added a functional assessment of mobility decrease, which might help to elucidate the patterns of loss of ADLs. Early detection of quantified mobility decrease might be beneficial for recognizing which individuals are most in need of intervention to reduce their risk of increasing care level. This study also suggests the possibility that the Locomotive Syndrome Risk Test might be a useful tool for monitoring mobility decrease among elderly with disability, which would be essential for taking early measures against aggravation of mobility decrease.

This study has several limitations. First, this is a cross-sectional study of rather few elderly with disability. Thus, further large-scale longitudinal studies will be needed to make any conclusions on age-independency in elderly with disability. Second, our data did not include other possible factors influencing mobility, such as presence of severe complications, recent hospitalizations, or socioeconomic status (e.g. marital status, occupation, and living alone or not). Further investigation will be needed to conclusively determine the effects of age and mobility decrease on elderly with disability. Third, there is potential selection bias in the independent community dwellers because they were recruited from people who visited a medical center for a periodic medical check-up.

## Conclusion

Among elderly with disability, the three test scores in the Locomotive Syndrome Risk Test were age-independent and worsened with severity of disability; conversely, in independent community dwellers, the three test scores were age-dependent. Further investigation will be needed to clarify the precise predictors of mobility decrease among elderly adults who have already disability.

## Abbreviations

GLFS: Geriatric locomotive function scale
JOA: Japanese Orthopaedic Association
LTCI: Long-term care insurance
